# Exploring the effect of differing centre hydration and anti-emetic policies on acute gastrointestinal and renal toxicities in the De-ESCALaTE trial

**DOI:** 10.1038/s44276-025-00132-7

**Published:** 2025-04-23

**Authors:** Anthony Kong, Matthew Hazell, Gulnaz Iqbal, Janet Dunn, Hisham Mehanna

**Affiliations:** 1 Comprehensive Cancer Centre, King’s College London, Guy’s Campus, London, UK; 2 Warwick Clinical Trials Unit, Warwick Medical School, University of Warwick, Coventry, CV4 7AL UK; 3 Institute of Head and Neck Studies and Education, Robert Aitken Building, University of Birmingham, Birmingham, UK

## Abstract

**Background:**

The De-ESCALaTE trial confirmed the superiority of cisplatin over cetuximab in combination with radiotherapy for the treatment of low risk HPV+ oropharyngeal cancer (HPV + OPC). However, there were concerns about certain toxicities with the use of cisplatin, in particular nausea, vomiting, dehydration and renal toxicities.

**Methods:**

The De-ESCALaTE trial collected data on several centre level policies on hydration and anti-emetic use. Univariable and backwards stepwise multivariable logistic regression models were used to model the association between centre level policy variables and severe adverse events (SAEs) of interest and severe (grade 3–5) acute toxicities of interest. In addition, the predictive performance of each model was assessed.

**Results:**

Centre level policies including the use of a triple anti-emetics regimen pre and post chemotherapy, increased volumes of IV fluids given before and during cisplatin chemotherapy as well as oral fluids advised post chemotherapy, were all associated with a reduced odds of SAEs of interest. Only a policy to give diuretics was associated with a reduction of severe (grade 3–5) acute toxicities of interest.

**Conclusions:**

For centres with HPV + OPC patients undergoing chemoradiation, we recommend the use of specific hydration and anti-emetic policies to reduce the rates of relevant SAEs and severe acute toxicities.

## Background

Concurrent cisplatin with radiation has been shown to increase survival compared to radiation alone and is considered standard of care for patients with locally advanced head and neck squamous cell carcinoma (HNSCC) undergoing radical chemoradiotherapy [1, 2]. More recently, the De-ESCALaTE (ISRCTN33522080) [3] and the RTOG 1016 [4] trials confirmed the superiority of cisplatin over cetuximab in combination with radiotherapy for the treatment of low risk Human papillomavirus positive oropharyngeal cancer (HPV + OPC) in terms of recurrence and overall survival.

These studies also showed that there were no differences between the overall all-grade and severe toxicity rates between the cisplatin and cetuximab arms, but the spectrum of toxicities differed between the two treatment arms [3, 4]. In the De-ESCALaTE trial, the most common acute severe (grade 3 to 5) toxicities associated with concurrent cisplatin were gastrointestinal-related, and there were also more renal, haematological, and metabolic toxicities compared to concurrent cetuximab. The most common causes of Serious Adverse Events (SAEs) for cisplatin were vomiting (30%), nausea (28%) and dehydration (16%), in contrast with oral mucositis (13%) and vomiting (13%) for concurrent cetuximab [5]. In addition, acute kidney injury was the cause of 10% of SAEs for cisplatin but only 5% for cetuximab. Within the De-ESCALaTE trial the overall mean number of acute severe toxicities grade 3–5 per patient reported for the cisplatin arm was 4.40 (95% CI 3.85; 4.95) [3].

The regimens for cisplatin (100 mg/m^2^ given intravenously three weekly) and radiotherapy (70 Gy in 35 fractions over 7 weeks) were mandated in the De-ESCALaTE trial. However, participating centres utilized their own differing policies in relation to hydration and anti-emetics used before, during and after cisplatin chemotherapy. These centre-level hydration and anti-emetic policies may play a role in mitigating nausea, vomiting, dehydration and acute renal toxicities among patients undergoing chemoradiation with cisplatin for the treatment of low risk human papillomavirus positive oropharyngeal cancer (HPV + OPC).

In this pre-specified analysis, we examined the effect of centre level policies on hydration and anti-emetic use on the overall incidence of SAEs reporting toxicities of interest (nausea, vomiting, dehydration and renal toxicities) and severe (grade 3–5) acute toxicities of interest (nausea, vomiting, dehydration and renal toxicities) in the De-ESCALaTE trial cisplatin arm.

## Methods

### Trial design

The De-ESCALaTE trial was an international, unblinded, multicentre, randomized controlled trial. Eligible patients were randomly assigned to undergo intensity-modulated radiotherapy (70 Gy in 35 fractions) with either three doses of IV cisplatin 100 mg/m^2^ on days 1, 22 and 43 of radiotherapy (control) or IV cetuximab 400 mg/m^2^ loading dose one week prior to radiotherapy followed by seven weekly infusions of 250 mg/m^2^ during radiotherapy (intervention) [5].

### Patients

Patients were recruited by their treating clinicians and all provided written informed consent. Study participants had to be low-risk HPV + OPC, non-smokers or had a lifetime self-reported smoking history of <10 pack-years, and had a tumour sample that was positive on p16 immunohistochemistry. Patients had to have an Eastern Cooperative Oncology Group (ECOG) performance status (PS) 0 or 1, and adequate renal, haematological and hepatic function for cisplatin-based, curative chemoradiotherapy. In addition, only patients who were eligible for De-ESCALaTE and had received at least one cycle of cisplatin were included in this analysis.

### Data collection

Data collected within the De-ESCALaTE study included baseline characteristics, the total dosage of cisplatin received and the number of doses received by the patient, as well as the doses of radiotherapy and any radiotherapy delays or interruptions.

Centres who participated in the De-ESCALaTE trial were asked to complete details about their standard anti-emetic and hydration policies and requested to send a template copy of their prescription for these to the trial coordinating centre to review. Each centre policy was coded into a spreadsheet and linked to the De-ESCALaTE SAE trial data. The following information about the centres’ regimens for hydration and anti-emetics were collected: whether there was a pre-hydration policy, the use of a diuretic policy (frusemide or mannitol), the amount of intravenous fluids given before, during and after chemotherapy, whether oral fluid after chemotherapy was advised, and type of antiemetic regimen prescribed after chemotherapy administration, including if a triple antiemetic regimen with a NK1 receptor antagonist (e.g. aprepitant or fosaprepitant) in addition to steroids and a serotonin 5-HT3 antagonist (ondansetron or granisetron) was given before and after chemotherapy, and whether any other anti-emetics (e.g. cyclizine, domperidone, metoclopramide) were prescribed after chemotherapy (see Supplementary Table 1 for an explanation of centre level hydration and anti-emetic policies). These centre level policies on hydration and anti-emetic use were the predictive variables of interest.

### Assessments and outcomes

Patients were assessed for treatment response 12 weeks after radiotherapy completion by clinical examination, and by CT, MRI scan or PET-CT scan. Follow-up consisted of clinical examination and toxicity assessment with the Common Toxicity Criteria Adverse Events (CTCAE) version 4), monthly in the first year and bimonthly in the second year, for at least 24 months from the end of completion.

For the purpose of this analysis, the cisplatin-induced toxicity events of interest were defined as any patient who had at least one toxicity recorded as nausea, vomiting, dehydration or renal. The first outcome was an SAE with at least one of the toxicities of interest at any grade recorded (TI-SAE). The second outcome was a severe (grade 3–5) acute toxicity of interest. Toxicities were classified as acute if they first appeared during or up to three months after treatment. Multiple occurrences of events of a single toxicity type within an analysis time period were counted as a single event.

### Statistical analysis

Univariable analyses were performed (using chi-square tests for categorical variables and t-tests for continuous variables) to explore the association between potential predictive centre level policy variables and the two outcomes of interest: [1] SAE with at least one of the toxicities of interest at any grade recorded and [2] a severe (grade 3–5) acute toxicity of interest.

Backward stepwise multivariable logistic regression models were then used to model the association between potential predictive variables and the two toxicity related outcomes of interest. These models included all centre level predictive variables (policies on pre-hydration, diuretics, the amount of intravenous (IV) fluids given before, during and after chemotherapy, whether oral fluid after chemotherapy was advised, and type of antiemetic regimen prescribed after chemotherapy administration, and whether any other anti-emetics were prescribed after chemotherapy) and iteratively excluded those not deemed to be associated to the outcome (*p* value < 0.05). The odds ratio and coefficient, and their 95% confidence interval (95% CI), as well as *p*-values from Wald tests were reported. These models were repeated including all relevant individual baseline demographic and clinical variables (total cisplatin dose, number of cisplatin doses, age, gender, clinical T stage, clinical N stage, ever smoked, type of radiotherapy, whether received percutaneous endoscopic gastrostomy feeding tube, whether received a neck dissection, the subsite of cancer and ECOG score) to control for confounding due to potential differences in participants between centres.

The predictive performance of the models was assessed by calibration and discrimination using receiver operator curves and bootstrapping [6]. The models developed within the bootstrapped dataset, which included 215 datasets each with a sample size of 161, were then applied to the original sample and the values of the performance within this dataset were known as performance values [7]. The 95% CIs for these values was determined using the percentile method. The performance of the final model was also assessed in the presence of baseline characteristics.

### Study conduct

HM and JD with the Trial Management Group designed the De-ESCALaTE trial, which was co-ordinated by Warwick Clinical Trials Unit, the details of which can be found in the previous publication [5]. AK was the clinical adviser in the De-ESCALaTE trial and assessed the submitted SAE reports during the study. Full protocol of the De-ESCALaTE trial can be accessed on https://www.inhanse.org/patient-research/de-escalate/.

### Role of funding source

The study funder had no role in design, collection, analysis or write up of the study. There was no commercial support for the study.

## Results

Between October 2012 and October 2016, 334 patients from 32 centres (31 treatment centres) were recruited into the De-ESCALaTE trial. Of the 166 patients allocated to receive cisplatin, 5 did not start treatment and were excluded from this analysis. Information about hydration and anti-emetic policies were obtained from each centre.

### Hydration and anti-emetic policies

All centres had a policy to give pre-chemotherapy hydration (prehydration) to patients, hence this variable was not included in subsequent analyses, and 87.1% had a policy to give diuretics (Table 1). The majority of centres (87.1%) gave up to 2 litres (L) of IV fluids before and during chemotherapy, with the rest giving 2.5 L or 3 L. 58.1% of centres gave more than 1 L of IV fluids after chemotherapy, rather than 1 L or less. Only 22.6% of centres advised patients to drink additional fluids orally after chemotherapy. Additionally, 54.8% of centres gave triple regimen anti-emetics before and after chemotherapy, with 80.6% also giving other anti-emetics.

**Table 1:** Hydration and anti-emetics policy for each of the 31 sites.

Centre policy information	Total (*N* = 31)
Hydration summaries	
Prehydration	
No	0 (0%)
Yes	31 (100%)
Diuretics	
No	4 (12.9%)
Yes	27 (87.1%)
Pre & during chemo amount of IV fluids	
1.5 or 2 litres	27 (87.1%)
2.5 or 3 litres	4 (12.9%)
Post chemo amount of IV fluids	
1 litre	13 (41.9%)
More than 1 litre	18 (58.1%)
Oral fluids advised	
No	24 (77.4%)
Yes	7 (22.6%)
Anti-emetics summaries	
Triple regimen pre and post chemotherapy	
No	13 (41.9%)
Yes	17 (54.8%)
Unknown	1 (3.2%)
Other anti-emetics after chemotherapy	
No	5 (16.1%)
Yes	25 (80.6%)
Unknown	1 (3.2%)

### Patient characteristics and treatment

Table 2 presents the patient baseline characteristics, disease status and treatment information as well as the anti-emetics and hydration policy utilised by the participating centres split by the toxicity outcomes. The mean age was 57.1 years. The majority of patients were male (80.1%); had T1-T2 and N2-N3 disease (65.3% and 75.5%, respectively); and had cancers of the tonsil (65.8%); were of ECOG PS 0 (87%); and had never smoked (55.3%).

**Table 2:** Univariable analysis assessing patient baseline characteristics and predictor variables by SAEs and by severe acute toxicities of interest.

Variables	Overall	Serious adverse events of interest			Severe acute toxicities of interest		
		No	Yes	*P*	No	Yes	*P*
		*N* = 83	*N* = 78		*N* = 89	*N* = 72	
Baseline characteristics							
Age^a^							
Mean (SD)	57 (8)	58 (8)	56 (8)		59 (8)	55 (8)	
Min, max	34, 76	34, 76	36, 75	0.14	34, 76	36, 75	<0.01
Gender							
Male	129 (80.1%)	68 (52.7%)	61 (47.3%)		74 (83.1%)	55 (76.4%)	
Female	32 (19.9%)	15 (46.9%)	17 (53.1%)	0.55	15 (16.9%)	17 (23.6%)	0.29
Clinical variables							
Clinical T-Stage							
T1	41 (25.5%)	20 (48.8%)	21 (51.2%)		21 (23.6%)	20 (27.8%)	
T2	64 (39.8%)	36 (56.3%)	28 (43.8%)		38 (42.7%)	26 (36.1%)	
T3	25 (15.5%)	12 (48.0%)	13 (52.0%)		12 (13.5%)	13 (18.1%)	
T4	31 (19.3%)	15 (48.4%)	16 (51.6%)	0.82	18 (20.2%)	13 (18.1%)	0.72
Clinical N-Stage							
N0	15 (9.3%)	7 (46.7%)	8 (53.3%)		7 (7.9%)	8 (11.1%)	
N1	24 (14.9%)	13 (54.2%)	11 (45.8%)		17 (19.1%)	7 (9.7%)	
N2a	21 (13.0%)	10 (47.6%)	11 (52.4%)		11 (12.4%)	10 (13.9%)	
N2b	79 (49.1%)	45 (57.0%)	34 (43.0%)		43 (48.3%)	36 (50.0%)	
N2c	21 (13.0%)	8 (38.1%)	13 (61.9%)		10 (11.2%)	11 (15.3%)	
N3	1 (0.6%)	0 (0%)	1 (100.0%)	0.57	1 (1.1%)	0 (0.0%)	0.52
Ever smoked?							
No	89 (55.3%)	47 (52.8%)	42 (47.2%)		50 (56.2%)	39 (54.2%)	
Yes	72 (44.7%)	36 (50.0%)	36 (50.0%)	0.72	39 (43.8%)	33 (45.8%)	0.80
Median pack years (IQR)^a^	6.5 (3–13)	7 (3–17.5)	6 (3–10)	0.14	6.0 (2.0–20.0)	7.0 (3.0–10.0)	0.12
Radiotherapy							
Unilateral	33 (20.5%)	15 (45.5%)	18 (54.5%)		14 (15.7%)	19 (26.4%)	
Bilateral	128 (79.5%)	68 (53.1%)	60 (46.9%)	0.43	75 (84.3%)	53 (73.6%)	0.10
Received PEG use							
No	36 (22.4%)	16 (44.4%)	20 (55.6%)		19 (21.3%)	17 (23.6%)	
Yes	125 (77.6%)	67 (53.6%)	58 (46.4%)	0.33	70 (78.7%)	55 (76.4%)	0.73
Received Neck dissection							
No	149 (92.6%)	79 (53.0%)	70 (47.0%)		83 (93.3%)	66 (91.7%)	
Yes	12 (7.4%)	4 (33.3%)	8 (66.7%)	0.19	6 (6.7%)	6 (8.3%)	0.70
Subsite							
Base of Tongue	53 (32.9%)	29 (54.7%)	24 (45.3%)		32 (36.0%)	21 (29.2%)	
Tonsil	106 (65.8%)	52 (49.1%)	54 (50.9%)		56 (62.9%)	50 (69.4%)	
Other	2 (1.2%)	2 (100.0%)	0 (0%)	0.31	1 (1.1%)	1 (1.4%)	0.66
ECOG							
0	140 (87.0%)	72 (51.4%)	68 (48.6%)		76 (85.4%)	64 (88.9%)	
1	21 (13.0%)	11 (52.4%)	10 (47.6%)	0.94	13 (14.6%)	8 (11.1%)	0.51
Centre level hydration summaries							
Pre-hydration							
No	0 (0%)	0 (0%)	0 (0%)		0 (0%)	0 (0%)	
Yes	161 (100.0%)	83 (51.6%)	78 (48.4%)	N/A	89 (100.0%)	72 (100.0%)	N/A
Diuretics							
No	26 (16.2%)	8 (30.8%)	18 (69.2%)		7 (7.9%)	19 (26.4%)	
Yes	135 (83.8%)	75 (55.6%)	60 (44.4%)	0.02	82 (92.1%)	53 (73.6%)	<0.01
Pre & during chemo amount of IV fluids							
1.5 or 2 litres	141 (87.6%)	67 (47.5%)	74 (52.5%)		74 (83.1%)	67 (93.1%)	
2.5 or 3 litres	20 (12.4%)	16 (80.0%)	4 (20.0%)	<0.01	15 (16.9%)	5 (6.9%)	0.06
Post chemo amount of IV fluids							
1 litre	73 (45.3%)	33 (45.2%)	40 (54.8%)		37 (41.6%)	36 (50.0%)	
>1 litre	88 (54.7%)	50 (56.8%)	38 (43.2%)	0.14	52 (58.4%)	36 (50.0%)	0.29
Oral fluids advised							
No	110 (68.3%)	52 (47.3%)	58 (52.7%)		55 (61.8%)	55 (76.4%)	
Yes	51 (31.7%)	31 (60.8%)	20 (39.2%)	0.11	34 (38.2%)	17 (23.6%)	0.05
Centre level anti-emetics summaries							
Triple regimen pre and post chemo							
No	69 (42.9%)	32 (46.4%)	37 (53.6%)		16 (18.2%)	12 (16.7%)	
Yes	92 (57.1%)	51 (55.4%)	41 (44.6%)	0.26	72 (81.8%)	60 (83.3%)	0.71
Other anti-emetics							
No	28 (17.4%)	16 (57.1%)	12 (42.9%)		2 (2.2%)	4 (5.6%)	
Yes	132 (82.0%)	66 (50.0%)	66 (50.0%)	0.49	33 (37.1%)	22 (30.6%)	0.80
Missing	1 (0.6%)	1 (100.0%)	0 (0%)		54 (60.7%)	46 (63.9%)	
Dose information							
Number of doses received							
1	6 (3.7%)	2 (33.3%)	4 (66.7%)		1 (16.7%)	5 (83.3%)	
2	55 (34.2%)	28 (50.9%)	27 (49.1%)		9 (16.4%)	46 (83.6%)	
3	100 (62.1%)	53 (53%)	47 (47%)	0.64	20 (20.0%)	80 (80.0%)	0.42
Total dose received (mg/m^2^)^a^							
N	161	83	78		30	131	
Median IQR	200 200, 300	200 200–300	200 200–300	0.10	200 200, 300	200 200, 300	0.58
Min, Max	100, 302.5	100, 302.5	100, 300		100.0, 302.5	100.0, 300.0	

^a^*P*-value calculated using t-test instead of Chi^2^ test.

The median duration from randomisation to radiotherapy start was 13 days (interquartile range 11–17) and did not differ between groups. Only 5.0% of patients received a dose of less than 70 Gy in the cisplatin arm (Supplementary Table 2). Radiotherapy interruptions or modification occurred in 8.7% patients receiving cisplatin.

Of the 161 patients who received cisplatin, 62 (38.5%) received all three cycles of cisplatin, 83 (51.6%) received two cycles and 16 (9.9%) received only one cycle (Supplementary Table 3). Of those that received only one or two doses of cisplatin, 13 (13.1%) received one or two doses of carboplatin instead. The median total cisplatin dose received was 200 mg/m^2^ (IQR 200,300) and only 16% received less than 200 mg/m^2^ in total. The main reasons for discontinuation or reduction in cisplatin dose were myelosuppression, oral/GI toxicity or nausea and vomiting. The specific reasons of switching from cisplatin to carboplatin due to different toxicities (such as nephrotoxicity, ototoxicity or neurotoxicity) was not available.

### Univariable analyses

There were 163 SAEs recorded on the cisplatin arm. Of these 102 SAEs (179 events from 78 patients) were reported to have suffered at least one of the four toxicities of interest (any grade) due to 67 (37%) nausea, 66 (37%) vomiting, 28 (17%) dehydration and 18 (10%) acute kidney problems (Supplementary Table 4).

There were 709 (141 patients) severe (grade 3–5) acute toxicities reported in the cisplatin arm of De-ESCALaTE trial, of which 120 (72 patients) were toxicities of interest (Supplementary Tables 5 and 6). The mean number of events of interest per patient was 1.67 (standard deviation = 0.65).

In the univariable analysis no baseline patient characteristics were associated with SAEs of interest, and only age was associated with severe acute toxicities of interest. Despite variation in the number of doses of concurrent cisplatin therapy with radiotherapy there was no association between the number of doses of cisplatin with either toxicity outcome.

Univariable analyses demonstrated evidence for an association between policies using diuretics (*p* = 0.02) and the amount of IV fluids given before and during chemotherapy (*p* < 0.01) with TI-SAEs of any grade. However, there was no evidence for a relationship between other centre level policies and this outcome.

For severe acute toxicities of interest, there was evidence for an association with the centre level policies on the use of diuretics (*p* < 0.01) and weak evidence for an association with advice for oral fluids to be given after chemotherapy (*p* = 0.05) and the amount of IV fluids given before and during chemotherapy (*p* = 0.06). No other centre level hydration or anti-emetic policy variables were associated with this outcome.

### Models to predict SAEs where variables of interest are mentioned (TI-SAEs) at any grade

Following the univariable analyses, we proceeded to explore the anti-emetic and hydration policy variables with a backward stepwise multivariable logistic regression for TI-SAEs at any grade. Advised oral fluids (OR: 0.43, 95% CI 0.19; 0.98), IV fluids given before, during (OR: 0.14, 95% CI 0.04; 0.50) and after (OR: 0.51, 95% CI 0.25; 1.04) chemotherapy, and triple anti-emetics regimens used during, and post chemotherapy (OR: 0.41, 95% CI 0.19; 0.89) were associated with TI-SAEs at any grade (Table 3a). There was no evidence for an association with the use of diuretics in this multivariable model, despite the relationship in univariable analyses. Repeating the model with the four identified centre level policy variables as well as potentially confounding baseline factors (total cisplatin dose, number of cisplatin doses, age, gender, clinical T stage, clinical N stage, ever smoked, type of radiotherapy, whether received percutaneous endoscopic gastrostomy feeding tube, whether received a neck dissection, the subsite of cancer, and ECOG score), resulted in there no longer being an association between post chemo amount of IV fluids and the outcome, but did not meaningfully alter the associations with other centre level hydration and anti-emetic policy variables (Supplementary Table 7).

**Table 3:** Initial model using stepwise backward multivariable logistic regression to predict SAEs where a variable of interest was recorded (any grade).

Centre level policy		Coefficient (95% CI)	OR (95% CI)	*P*-value
Oral fluids advised	No (reference)			
	Yes	−0.84 (−1.66; −0.21)	0.43 (0.19; 0.98)	0.04
Pre & during chemo amount of IV fluids	1.5 or 2 litres (reference)			
	2.5 to 3 litres	−1.97 (−3.25; −0.69)	0.14 (0.04; 0.50)	<0.01
Triple regimen anti-emetics	No (reference)			
	Yes	−0.89 (−1.65; −0.12)	0.41 (0.19; 0.89)	0.02
Post chemo amount of IV fluids	1 litre (reference)			
	More than 1 litre	−0.66 (−1.39; 0.04)	0.51 (0.25; 1.04)	0.06

The predictive performance of the model was assessed by calibration and discrimination. The area under the ROC curve was 0.71 (95% CI: 0.63–0.79) which suggests that there was fair discrimination. The optimism-corrected estimate of performance was 0.66 (95% CI 0.57; 0.72). Similar results were shown when the model to predict SAEs using stepwise backward multivariable logistic regression was performed in the presence of baseline characteristics (ROC: 0.76, 95% CI 0.68; 0.83).

### Models to predict severe (grade 3–5) acute toxicities of interest

The only anti-emetic and hydration policy variable associated with severe acute (grade 3–5) toxicities of interest (with or without being reported as SAEs) in the backward stepwise multivariable logistic regression model was the use of diuretics (OR 0.24, 95% CI 0.09;0.61) (Table 4). Advice for oral fluids to be given after chemotherapy and the amount of IV fluids given before and during chemotherapy which demonstrated weak evidence of an association with the outcome in univariable analyses, did not have a relationship with the outcome in this model. Adding baseline characteristics to the model did not meaningfully change the relationship (Supplementary Table 8). The area under the ROC curve, which estimates the predictive performance of the model, was 0.59 (95% CI 0.53; 0.65) which represents very weak discrimination from this model. The optimism-corrected estimate of performance was 0.58 (95% CI 0.54; 0.62). In the presence of baseline variables, the model discrimination was improved (ROC: 0.75, 95% CI 0.68; 0.83).

**Table 4:** Initial model using stepwise backward multivariable logistic regression to predict severe toxicities.

Centre level policy		Coefficient (95% CI)	OR (95% CI)	*P*-value
Use of diuretics	No (reference)			
	Yes	−1.42 (−2.36; −0.49)	0.24 (0.09; 0.61)	<0.01

When sensitivity analyses were conducted investigating all severe acute toxicities (grade 3–5) among study participants (141/161), no centre level hydration or anti-emetic policies were associated with this outcome.

## Discussion

This analysis shows that local hydration and anti-emetics policies for cisplatin treatment differed between participating centres in the De-ESCALaTE trial. The use of a triple anti-emetics regimen, 2.5 to 3 litres of IV fluids given before and during cisplatin chemotherapy as well as oral fluids advised post chemotherapy, were associated with a reduced incidence of SAEs with toxicities of interest (nausea, vomiting, dehydration and renal toxicities) of any grade. In addition, centres which had a policy to use diuretics had a lower incidence of severe (grade 3–5) acute toxicities of interest (nausea, vomiting, dehydration and renal toxicities).

The most widely accepted schedule of concomitant cisplatin with radiotherapy for locally advanced HNSCC has been 100 mg/m^2^ given on a three-weekly basis, which was also the regimen used in the De-ESCALaTE trial [3]. A phase III randomized non-inferiority trial of cisplatin 30 mg/m^2^ once a week compared with cisplatin 100 mg/m^2^ once every 3 weeks in locally advanced HNSCC conducted in India, demonstrated that once-every-week cisplatin at 30 mg/m^2^ was shown to have an inferior locoregional control albeit with less acute grade 3–5 toxicities than the three weekly cisplatin regimen [5]. However, a subsequent phase II/III JCOG1008 trial comparing weekly cisplatin (40 mg/m^2^) with 3-weekly cisplatin (100 mg/m^2^) showed that weekly cisplatin was noninferior to 3-weekly cisplatin for overall survival and resulted in less frequent ≥Grade 3 neutropenia, infection, renal and hearing impairment in high-risk HNSCC patients undergoing post-operative chemoradiotherapy [8]. While some cancer centres have changed their concomitant cisplatin chemotherapy with radical and/or postoperative radiotherapy to a weekly regimen, the three-weekly cisplatin regimen at 100 mg/m^2^ remains the standard of care at many centres [9].

A high dose of cisplatin (≥50 mg/m^2^) has a high emetic risk with 90% frequency of emesis and has the dose-limiting toxicity of nephrotoxicity [10]. Therefore, its administration usually requires hydration and use of antiemetic agents, often necessitating inpatient overnight stay. However, studies have assessed the use of shorter durations of hydration with lower hydration volumes to facilitate outpatient ambulatory delivery of cisplatin [10]. In addition, other strategies to minimize nephrotoxicity includes magnesium and diuretics such as mannitol in the hydration protocol. This is because magnesium depletion increases the severity of platinum-induced nephrotoxicity [11–13] and mannitol has been used to reduce the urine concentration of cisplatin to decrease its nephrotoxicity [14].

In a retrospective study, patients with lung cancer and other cancers were treated with cisplatin at a dose of ≥60 mg/m^2^ using a short hydration protocol consisting of a maximum of 2250 ml of hydration with mannitol and magnesium supplementation over a period of less than 5 h on Day 1 or a conventional hydration protocol consisting of a maximum of 2600 ml of hydration over less than 8 h on Day 1 with pre- and post-hydration for three days [15]. It was shown that ≥grade 1 elevated serum creatinine level was less frequent in the group receiving the short hydration protocol than in the group receiving conventional hydration [15]. In two other prospective Japanese studies, the short hydration with cisplatin was shown to be safe without severe renal toxicities in regimens containing cisplatin (75 mg/m^2^) for patients with lung cancer [16, 17]. However, the safety of the short hydration policy with concurrent high-dose three weekly cisplatin and radiation in HNSCC has not been formally assessed.

This novel observational study using existing clinical trial data has strengths including detailed information on centre level policy for hydration and antiemetic use, high-quality data on relevant baseline and clinical characteristics for participants, and no loss to follow up. In addition, the use of bootstrapping increased the stability of the calibration and discrimination estimates for the prediction models [18]. However, these results should be interpreted in light of certain limitations. Data on patient level hydration and anti-emetic use would have strengthened the conclusions, as use likely differs within centres depending on a patient’s clinical indication and a clinician’s preference. For example, for individuals using a PEG, a centre level policy advising patients to drink additional fluids orally after chemotherapy would not be relevant. This means that the ecological fallacy may be introduced if extrapolating these centre level findings to the individual [19]. Additionally, stepwise regression models can produce biased regression coefficients, CIs and *p* values [20]. Therefore, multivariable regression models were produced for the two outcomes with all centre level predictor variables, with and without all baseline variables (Supplementary Tables 9–12). In the presence of baseline characteristics, use of oral fluids, IV fluids given pre and during chemo, and use of diuretics were weakly associated with severe acute toxicities of interest (grade 3–5), but the use of a triple anti-emetics regimen pre and post chemotherapy, increased volumes of IV fluids given before and during cisplatin chemotherapy as well as oral fluids advised post chemotherapy were still associated with SAEs with toxicities of interest of any grade.

Unlike nephrotoxicity, there has been no evidence to suggest that hydration or any other approved preventive treatments are effective against ototoxicity and/or neurotoxicity induced by cisplatin [21]. It would have been interesting to assess different policies for discontinuing cisplatin or switching to carboplatin due to ototoxicity or neurotoxicity in addition to nephrotoxicity across different centres. However, this data was not available at the time of analysis and it was also out of scope of our research plan in investigating the effect of differing centre hydration and anti-emetic policies on acute gastrointestinal and nephrotoxicity toxicities in the De-ESCALaTE trial. This could be a potential interesting area of future study to assess variable practice in discontinuing cisplatin related to ototoxicity and/or neurotoxicity in addition to nephrotoxicity across different centres in the UK.

In conclusion, we recommend the use of a triple anti-emetic regimen, adequate hydration of 2.5–3 litres before and during chemotherapy as well as advising patients to take oral fluids and diuretics when giving concomitant cisplatin (100 mg/m^2^ three-weekly) with radiotherapy for locally advanced HPV+ head and neck cancers. These measures may reduce SAEs including hospitalization, life-threatening complication, death, significant disability/incapacity or permanent impairment/damage related to nausea, vomiting, dehydration and/or acute renal injury.

## Supplementary information

Supplementary Information

## Data Availability

De-identified participant data and the data dictionary will be available along with the study protocol and statistical analysis plan from Jan 1, 2020, onwards. Please email G.Iqbal@warwick.ac.uk. Data will not include name, address, hospital number or date of birth, or any other identifying data. The data will be accompanied by metadata which gives a complete explanation of the data fields, the definition, the standards used such as TNM staging, and the units used. The data will be shared through custodianship by the principal investigator. A data access committee will be convened and will comprise the principal investigator and two other co-investigators. They will be responsible for assessing requests for data sharing about granting access. The data management committee will be responsible to the steering committee and requests for appeals will be made directly to the trial steering committee. The process for requesting data sharing will be as follows: the requestor will complete a two-page proforma requiring name and contact details of the requestor, the objectives of the study, the methodology, the expected outcome, the statistical analysis plan, whether the project will be a collaboration with the DeESCALaTE study organisers or will only acknowledge the study and its organisers, ethical committee approval, and funding and peer review details. The data sharing committee will meet at least twice a year to consider these requests. Urgent requests may be considered in between these meetings. In the event of a declined application, the requestors may lodge an appeal with the trial steering committee chairperson. The dataset will be stored with the principal investigator at the Institute of Head and Neck Studies and Education in the long term. The data will be available for public release from the time of publication of the main results of the study. Prior to that, access of the data may be considered in specific circumstances. After 5 years of publication of the result, the data may then be lodged with a data archiving facility. Requestors who are granted access to the data will be required to complete a data sharing agreement, which is based on the principles, content, and format published by the National Cancer Research Institute (NCRI).
