# Supplementary material for: Exploring the effect of differing centre hydration and anti-emetic policies on acute gastrointestinal and renal toxicities in the De-ESCALaTE trial
Source: BJC Rep. 2025 Apr 23;3:25. doi: 10.1038/s44276-025-00132-7 (PMC12019484; doi:10.1038/s44276-025-00132-7)
Supplement: Supplementary file 1 — Supplementary Information [file 44276_2025_132_MOESM1_ESM.docx]

**Exploring the effect of differing centre hydration and anti-emetic policies on acute gastrointestinal and renal toxicities in the De-ESCALaTE trial: A supplementary file**

Anthony Kong*^a^, Matthew Hazell^b^, Gulnaz Iqbal^b^, Janet Dunn^b*^, and Hisham Mehanna^c*^

^a^Comprehensive Cancer Centre, King’s College London, Guy’s Campus, London, United Kingdom

^b^Warwick Clinical Trials Unit, Warwick Medical School, University of Warwick, Coventry, CV4 7AL, United Kingdom.

^c^Institute of Head and Neck Studies and Education, Robert Aitken Building, University of Birmingham, Birmingham, United Kingdom.

^*^These authors contributed equally to this work.

Corresponding author: Hisham Mehanna. Institute of Head and Neck Studies and Education, Robert Aitken Building, University of Birmingham, Birmingham, United Kingdom. Email: [h.mehanna@bham.ac.uk](mailto:h.mehanna@bham.ac.uk). ORCID ID: 0000-0002-5544-6224.

**Table of contents**

[Supplementary Table 1: Explanation of centre level hydration and anti-emetic policies 3](#_Toc171070550)

[Supplementary Table 2: Amount of radiotherapy received for Cisplatin patients 4](#_Toc171070551)

[Supplementary Table 3: Number of cycles of cisplatin or doses of carboplatin received per patient 5](#_Toc171070552)

[Supplementary Table 4: Number of SAEs reporting toxicities of interest 6](#_Toc171070553)

[Supplementary Table 5: Number of severe acute toxic events by toxicity of interest. 7](#_Toc171070554)

[Supplementary Table 6: Number of severe acute toxic events per person. 8](#_Toc171070555)

[Supplementary Table 7: Final model to predict SAEs using stepwise backward multivariable logistic regression in the presence of baseline characteristics. Baseline variables not shown. Corresponds with Table 3. 9](#_Toc171070556)

[Supplementary Table 8: Final model to predict severe toxicities of interest using stepwise backward multivariable logistic regression in the presence of baseline characteristics. Baseline variables not shown. Corresponds with table 4. 10](#_Toc171070557)

[Supplementary Table 9: Multivariable logistic regression model for the association between SAEs with toxicities of interest and centre level policies on hydration and anti-emetics. 11](#_Toc171070558)

[Supplementary Table 10: Multivariable logistic regression model for the association between SAEs with toxicities of interest in the presence of baseline characteristics and centre level policies on hydration and anti-emetics. Baseline variables not shown. 12](#_Toc171070559)

[Supplementary Table 11: Multivariable logistic regression model for the association between SAEs with acute severe toxicities of interest and centre level policies on hydration and anti-emetics. 13](#_Toc171070560)

[Supplementary Table 12: Multivariable logistic regression model for the association between SAEs with acute severe toxicities of interest and centre level policies on hydration and anti-emetics in the presence of baseline characteristics and centre level policies on hydration and anti-emetics. Baseline variables not shown. 14](#_Toc171070561)

# Supplementary Table 1: Explanation of centre level hydration and anti-emetic policies

| **Centre Policy Information** | **Explanation** |  |
| --- | --- | --- |
|  |  |  |
| **Hydration Summaries** |  |  |
| Prehydration policy | The use of a prehydration policy (Yes/No) |  |
| Diuretics policy | The use of a diuretic policy (frusemide or mannitol) (Yes/No) |  |
| Pre & during chemo amount of IV fluids | The amount of intravenous (IV) fluids given before and during chemotherapy (1.5 or 2 litres/2.5 or 3 litres) |  |
| Post chemo amount of IV fluids | The amount of IV fluids after chemotherapy (1 litre/more than 1 litre) |  |
| Oral fluids advised | Whether oral fluids after chemotherapy were advised (Yes/No) |  |
| **Anti-emetics summaries** | |  |
| Triple regimen pre and post chemotherapy | If a triple antiemetic regimen (with a NK1 receptor antagonist (e.g. aprepitant or fosaprepitant) in addition to steroids and a serotonin 5-HT3 antagonist (ondansetron or granisetron)) was given before and after chemotherapy (Yes/ No) |  |
| Other anti-emetics after chemotherapy | Whether any other anti-emetics emetics (e.g. cyclizine, domperidone, metoclopramide) were prescribed after chemotherapy (Yes/ No) |  |

# Supplementary Table 2: Amount of radiotherapy received for Cisplatin patients

| **Radiotherapy dose (Gy)** | **Cisplatin+RT N (%)** |  |
| --- | --- | --- |
|  |  |  |
| 65<x<70 | 8 (5.0%) |  |
| 70 | 144 (89.4%) |  |
| 70<x<75 | 8 (5.0%) |  |
| >75 | 1 (0.6%) |  |
| Total | 161 |  |

# Supplementary Table 3: Number of cycles of cisplatin or doses of carboplatin received per patient

| **Number of cycles of cisplatin** | **Number of doses of carboplatin** | | | **Total** |
| --- | --- | --- | --- | --- |
|  | 0 | 1 | 2 |  |
| 1 | 7 (4.7%) | 4 (50%) | 5 (100%) | 16 (9.9 %) |
| 2 | 79 (53.4%) | 4 (50%) | - | 83 (51.6%) |
| 3 | 62 (41.9%) | - | - | 62 (38.5%) |
| Total | 148 | 8 | 5 | 161 |

# Supplementary Table 4: Number of SAEs reporting toxicities of interest

| **Number of toxic events per patient** | **Nausea** | **Vomiting** | **Dehydration** | **Acute kidney** | **Total** |
| --- | --- | --- | --- | --- | --- |
| 1 | 9 (13.4%) | 12 (18.2%) | 9 (32.1%) | 6 (33.3%) | 36 (20.1%) |
| 2 | 48 (71.6%) | 45 (68.2%) | 11 (39.3%) | 6 (33.3%) | 110 (61.4%) |
| 3 | 10 (14.9%) | 9 (13.6%) | 8 (28.6%) | 6 (33.3%) | 33 (18.4%) |
| Total | 67 | 66 | 28 | 18 | 179 |

# Supplementary Table 5: Number of severe acute toxic events by toxicity of interest.

| **System organ class level** | **Term Level** | **N (%)** |
| --- | --- | --- |
| Gastrointestinal disorders | Nausea | 57 (47.5) |
| Gastrointestinal disorders | Vomiting | 33 (27.5) |
| Metabolism & nutrition orders | Dehydration | 19 (15.8) |
| Renal and urinary disorders | Acute Kidney injury | 9 (7.5) |
| Renal and urinary disorders | Other | 2 (1.67) |
| Total |  | 120 (100) |

# Supplementary Table 6: Number of severe acute toxic events per person.

| **Number of severe acute toxic events** | **Frequency** |  |
| --- | --- | --- |
|  |  |  |
| 1 | 31 (43.0%) |  |
| 2 | 34 (47.2%) |  |
| 3 | 7 (9.7%) |  |
| Total | 72 (100%) |  |

# Supplementary Table 7: Final model to predict SAEs using stepwise backward multivariable logistic regression in the presence of baseline characteristics. Baseline variables not shown. Corresponds with Table 3.

| **Centre level policy** | | **Coefficient (95% CI)** | **OR (95% CI)** | **P-value** |
| --- | --- | --- | --- | --- |
| Oral fluids advised | No (reference) |  |  |  |
|  | Yes | -1.09 (-2.04; -0.14) | 0.34 (0.13; 0.87) | 0.02 |
| Pre & during chemo IV fluids | 1.5 or 2 litres (reference) | |  |  |
|  | 2.5 to 3 litres | -2.20 (-3.64; -0.77) | 0.11 (0.03; 0.46) | <0.01 |
| Triple regimen anti-emetics pre and post chemotherapy | No (reference) |  |  |  |
|  | Yes | -0.94 (-1.81; -0.07) | 0.39 (0.16; 0.93) | 0.03 |
| Post chemo amount of IV fluids | 1 litre (reference) |  |  |  |
|  | More than 1 litre | -0.41 (-1.24; 0.42) | 0.66 (0.29; 1.52) | 0.33 |

*OR – Odds ratio, CI – Confidence interval.*

# Supplementary Table 8: Final model to predict severe toxicities of interest using stepwise backward multivariable logistic regression in the presence of baseline characteristics. Baseline variables not shown. Corresponds with table 4.

| **Centre level policy** |  | **Coefficient (95% CI)** | **OR (95% CI)** | **P-value** |
| --- | --- | --- | --- | --- |
| Use of diuretics | No (reference) |  |  |  |
|  | Yes | -1.74 (-2.84; -0.64) | 0.18 (0.06; 0.53) | <0.01 |

*OR – Odds ratio, CI – Confidence interval.*

# Supplementary Table 9: Multivariable logistic regression model for the association between SAEs with toxicities of interest and centre level policies on hydration and anti-emetics.

| **Centre level policy** | | **OR (95% CI)** | **P-value** |
| --- | --- | --- | --- |
| Use of diuretics | No (reference) |  |  |
|  | Yes | 0.75 (0.27; 2.09) | 0.58 |
| Pre & during chemo IV fluids | 1.5 or 2 litres (reference) |  |  |
|  | 2.5 to 3 litres | 0.15 (0.03; 0.73) | 0.02 |
| Post chemo amount of IV fluids | 1 litre (reference) |  |  |
|  | More than 1 litre | 0.51 (0.24; 1.10) | 0.09 |
| Oral fluids | No (reference) |  |  |
|  | Yes | 0.42 (0.16; 1.09) | 0.07 |
| Triple regimen anti-emetics pre and post chemotherapy | No (reference) |  |  |
|  | Yes | 0.38 (0.16; 0.88) | 0.02 |
| Other antiemetics | No (reference) |  |  |
|  | Yes | 1.06 (0.28; 4.01) | 0.92 |

*OR – Odds ratio, CI – Confidence interval.*

# Supplementary Table 10: Multivariable logistic regression model for the association between SAEs with toxicities of interest in the presence of baseline characteristics and centre level policies on hydration and anti-emetics. Baseline variables not shown.

| **Centre level policy** | | **OR (95% CI)** | **P-value** |
| --- | --- | --- | --- |
| Use of diuretics | No (reference) |  |  |
|  | Yes | 0.88 (0.28; 2.84) | 0.84 |
| Pre & during chemo IV fluids | 1.5 or 2 litres (reference) |  |  |
|  | 2.5 to 3 litres | 0.12 (0.02; 0.70) | 0.02 |
| Post chemo amount of IV fluids | 1 litre (reference) |  |  |
|  | More than 1 litre | 0.64 (0.26; 1.57) | 0.33 |
| Oral fluids | No (reference) |  |  |
|  | Yes | 0.32 (0.11; 0.96) | 0.04 |
| Triple regimen anti-emetics pre and post chemotherapy | No (reference) |  |  |
|  | Yes | 0.36 (0.14; 0.93) | 0.04 |
| Other antiemetics | No (reference) |  |  |
|  | Yes | 1.26 (0.27; 5.86) | 0.77 |

*OR – Odds ratio, CI – Confidence interval.*

# Supplementary Table 11: Multivariable logistic regression model for the association between SAEs with acute severe toxicities of interest and centre level policies on hydration and anti-emetics.

| **Centre level policy** | | **OR (95% CI)** | **P-value** |
| --- | --- | --- | --- |
| Use of diuretics | No (reference) |  |  |
|  | Yes | 0.36 (0.13; 1.04) | 0.06 |
| Pre & during chemo IV fluids | 1.5 or 2 litres (reference) |  |  |
|  | 2.5 to 3 litres | 0.35 (0.08; 1.44) | 0.15 |
| Post chemo amount of IV fluids | 1 litre (reference) |  |  |
|  | More than 1 litre | 0.83 (0.40; 1.72) | 0.61 |
| Oral fluids | No (reference) |  |  |
|  | Yes | 0.50 (0.20; 1.26) | 0.61 |
| Triple regimen anti-emetics pre and post chemotherapy | No (reference) |  |  |
|  | Yes | 0.59 (0.27; 1.33) | 0.21 |
| Other antiemetics | No (reference) |  |  |
|  | Yes | 0.95 (0.27; 3.36) | 0.33 |

*OR – Odds ratio, CI – Confidence interval.*

# Supplementary Table 12: Multivariable logistic regression model for the association between SAEs with acute severe toxicities of interest and centre level policies on hydration and anti-emetics in the presence of baseline characteristics and centre level policies on hydration and anti-emetics. Baseline variables not shown.

| **Centre level policy** | | **OR (95% CI)** | **P-value** |
| --- | --- | --- | --- |
| Use of diuretics | No (reference) |  |  |
|  | Yes | 0.30 (0.85; 1.04) | 0.06 |
| Pre & during chemo IV fluids | 1.5 or 2 litres (reference) |  |  |
|  | 2.5 to 3 litres | 0.20 (0.37; 1.11) | 0.07 |
| Post chemo amount of IV fluids | 1 litre (reference) |  |  |
|  | More than 1 litre | 1.21 (0.48; 3.09) | 0.69 |
| Oral fluids | No (reference) |  |  |
|  | Yes | 0.32 (0.10; 0.98) | 0.05 |
| Triple regimen anti-emetics pre and post chemotherapy | No (reference) |  |  |
|  | Yes | 0.50 (0.19; 1.33) | 0.16 |
| Other antiemetics | No (reference) |  |  |
|  | Yes | 0.96 (0.18; 4.97) | 0.96 |

*OR – Odds ratio, CI – Confidence interval.*
